# Relative Telomere Length Is Associated With Age-Related Macular Degeneration in Women

**DOI:** 10.1167/iovs.63.5.30

**Published:** 2022-05-25

**Authors:** Adriana Koller, Caroline Brandl, Claudia Lamina, Martina E. Zimmermann, Monika Summerer, Klaus J. Stark, Reinhard Würzner, Iris M. Heid, Florian Kronenberg

**Affiliations:** 1 Institute of Genetic Epidemiology, Medical University of Innsbruck, Innsbruck, Austria; 2 Department of Genetic Epidemiology, University of Regensburg, Regensburg, Germany; 3 Department of Ophthalmology, University Hospital Regensburg, Regensburg, Germany; 4 Institute of Hygiene and Medical Microbiology, Medical University of Innsbruck, Innsbruck, Austria

**Keywords:** relative telomere length, age-related macular degeneration (AMD), mobile elderly population, diseases of late onset

## Abstract

**Purpose:**

Relative telomere length (RTL) is a biomarker for physiological aging. Premature shortening of telomeres is associated with oxidative stress, which is one possible pathway that might contribute to age-related macular degeneration (AMD). We therefore aimed to investigate the association between RTL and AMD in a well-characterized group of elderly individuals.

**Methods:**

We measured RTL in participants of the AugUR study using a multiplex quantitative PCR-based assay determining the ratio between the telomere product and a single-copy gene product (T/S ratio). AMD was assessed by manual grading of color fundus images using the Three Continent AMD Consortium Severity Scale.

**Results:**

Among the 2262 individuals 70 to 95 years old (627 with AMD and 1635 without AMD), RTL was significantly shorter in individuals with AMD compared to AMD-free participants. In age- and sex-adjusted logistic regression analyses, we observed an 8% higher odds for AMD per 0.1 unit shorter RTL (odds ratio [OR] = 1.08; 95% confidence interval [CI], 1.02–1.14; *P* = 0.005). The estimates remained stable when adjusted for smoking, high-density lipoprotein cholesterol, cardiovascular disease, diabetes, and hypertension. Interestingly, this association was only present in women (OR = 1.14; 95% CI, 1.06–1.23; *P* < 0.001), but not in men (OR = 1.01; 95% CI, 0.93–1.10; *P* = 0.76). A significant sex-by-RTL interaction on AMD was detected (*P* = 0.043).

**Conclusions:**

Our results show an association of RTL with AMD that was restricted to women. This is in line with altered reactive oxygen species levels and higher telomerase activity in women and provides an indication for a sex-differential pathway for oxidative stress and AMD.

Oxidative stress is a well-described pathway for many diseases, including age-related macular degeneration (AMD),^1^ a disease causing irreversible central vision loss in the elderly population of Western societies.^2^^,^^3^ In line with this, smoking and diet have been shown to play a role in AMD.^4^^,^^5^ Early AMD stages, characterized by drusen/drusenoid deposits and retinal pigment epithelium abnormalities, precedes late disease, defined by geographic atrophy of the retinal epithelium or neovascular consequences with scarring.^3^^,^^5^^–^^8^ In epidemiological studies, both early and late AMD are assessed and graded via standardized color fundus photography.^9^

Several studies showed that chronic inflammation and complement activation play a crucial role in the formation of drusen and the development of this disease.^10^^–^^12^ Moreover, inflammation, mitochondrial dysfunction, and oxidative stress are involved not only in physiological aging but also predominantly in AMD.^1^^,^^13^^–^^17^ Reactive oxygen species (ROS) and oxidative stress markers can be measured by, for example, liquid chromatography, spectrophotometry, or chemiluminescence assays and ELISA.^18^^,^^19^ However, DNA damage induced by high levels of ROS associated with oxidative stress affects particularly telomeric DNA. Hence, another approach to assess elevated oxidative stress levels is to investigate the telomere length, a potential marker for increased oxidative stress.^20^^–^^22^

Telomeres are non-coding, repetitive DNA sequences with a 5′-TTAGGG-3′ motif at the ends of linear chromosomes. These sequences reach lengths of 5 to 15 kb and are crucial for genome integrity by capping and protecting the genetic material.^14^^,^^23^ Telomeres, which are the longest at birth, shorten progressively with each cell replication as the DNA polymerase is unable to completely replicate the 3′ end of the lagging strand.^16^ At a certain point, known as the Hayflick limit,^24^ the shortened telomeres cause cell death or senescence. However, there are various other factors besides age and the associated summation of cell divisions that can induce premature shortening of the telomeres—for example, inflammation or increased oxidative stress.^14^^,^^17^^,^^20^^,^^25^^,^^26^ Telomere attrition beyond the physiological process that comes with aging is associated with an increased risk for mortality, cardiovascular disease, kidney disease, and type 2 diabetes (reviewed in References 14 and 27). Therefore, telomere length is not solely a biomarker for aging but also a potential predictor for diseases.^17^

Existing literature on the association between RTL and AMD is sparse,^28^^–^^30^ and consistent evidence for an association is missing. Conflicting results, mostly from small studies, have been reported, including a negative as well as a positive association between RTL and AMD^28^^,^^30^ or no association at all.^29^ Several questions remain unanswered, and especially large epidemiological studies with detailed ophthalmologic characterizations are needed.

In this study, we investigated the association of relative telomere length (RTL) with AMD and hypothesized that RTL might contribute to this disease. We measured RTL in >2000 participants 70 to 95 years old in the population-based AugUR study, including more than 600 individuals with AMD.

## Methods

### Study Population and Data Collection

The Altersbezogene Untersuchungen zur Gesundheit der University of Regensburg (AugUR) study was initiated to investigate genetic and non-genetic risk factors for age-related diseases. The background and design of this prospective study have been published previously,^31^ and more details are provided in the Supplementary Materials. Briefly, individuals living in or near Regensburg who were at least 70 years of age were invited to the study center, and all individuals willing to participate were included in the study (Supplementary Fig. S1). Via interview-based questionnaire and standardized medical examinations, we gained information on sociodemographic data, lifestyle factors, metabolic parameters, medication use, and general and ocular morbidities. Moreover, blood and urine samples were collected. In summary, this resulted in over 2400 participants being included in the ongoing AugUR study. Details on category definitions and variable measurements can be found in the Supplementary Materials. The AugUR study was approved by the Ethics Committee of the University of Regensburg, Germany (vote 12-101-0258). The study complies with the tenets of the Declaration of Helsinki and its later amendments. All participants provided informed written consent.

### AMD Assessment

Medical examinations for the AugUR study included color fundus photography of the central retina, which was used to assess AMD as described previously.^6^ Briefly, non-stereo color fundus photography was conducted using the automatized Digital Retinography System (DRS) camera (CenterVue, Padova, Italy) after administering a mild mydriasis as described previously.^6^ At least two images of each eye were acquired, and the central or central nasal fields of the retina were captured within a 45° view. Images for each eye were manually graded by applying the Three Continent AMD Consortium Severity Scale (no AMD, mild/moderate/severe early AMD, late AMD).^9^ The AMD status of a person was based on the eye with more advanced disease stage (worse eye definition). An experienced and trained ophthalmological consultant (C.B.) graded the color fundus images, and questionable findings were discussed with a second trained grader (ophthalmological consultant). Intergrader reliability was assessed via an independent grading by the second grader with a concordance of 95.3% and quadratic weighted kappa of 0.972.^6^

### RTL Measurements

To measure the RTL, quantitative PCR (qPCR) based on SYBR Green reagents was used.^32^ In contrast to our previous work on RTL,^33^^–^^35^ in this study we used the multiplex approach based on the method published by Cawthon.^32^ Details on DNA extraction and our slightly modified protocol for RTL measurements can be found in the Supplementary Materials. Briefly, the RTL was calculated via the ratio of the telomere product versus a single copy gene (T/S ratio). For this study, the quality criteria for measurement of RTL were met in all 2401 samples with available DNA.

### Statistical Analyses

All AugUR participants with available RTL measurements and AMD classifications were included in these analyses. From the 2401 individuals with available RTL measurements, 139 participants were excluded due to missing AMD classification status; therefore, the data of 2262 participants were included in the final analyses. All analyses were performed using R 4.0.4 (R Foundation for Statistical Computing, Vienna, Austria). A two-sided *P* < 0.05 was considered statistically significant.

First, we compared baseline characteristics between individuals with AMD and without AMD via unpaired *t*-tests and Wilcoxon rank-sum tests when comparing two groups, ANOVA and Kruskal–Wallis tests were used for comparisons among three groups, and χ² tests were used for categorical variables. Baseline characteristics are presented as mean ± standard deviation (SD) and quartiles or *N* (%). Odds ratios (ORs) were calculated via multinomial and logistic regression models using various adjustments to evaluate the association of RTL with AMD. Relative telomere length data were analyzed by a decline of 0.1 on the original data. We conducted multinomial regression analyses to compare the association of RTL on early or late AMD compared to no AMD. Because we noticed no major difference between early and late AMD, we combined the two groups into “any AMD.” Age, sex, and other possible confounders of the relationship between RTL and AMD were considered in the various regression models. Nonlinear splines for logistic and linear regression models were created including 95% confidence intervals (CIs).

We performed a priori power calculations for a logistic regression models with any AMD as the outcome (Supplementary Fig. S2). We had 80% power to detect ORs between 1.1 and 1.3 per standard deviation change of the RTL. Additionally, we tested the assumptions of the adjusted logistic regression model (e.g., multicollinearity, outliers and influential points via Cook's distance and linearity of independent variables).

## Results

### Participant Characteristics

**Figure 1.**
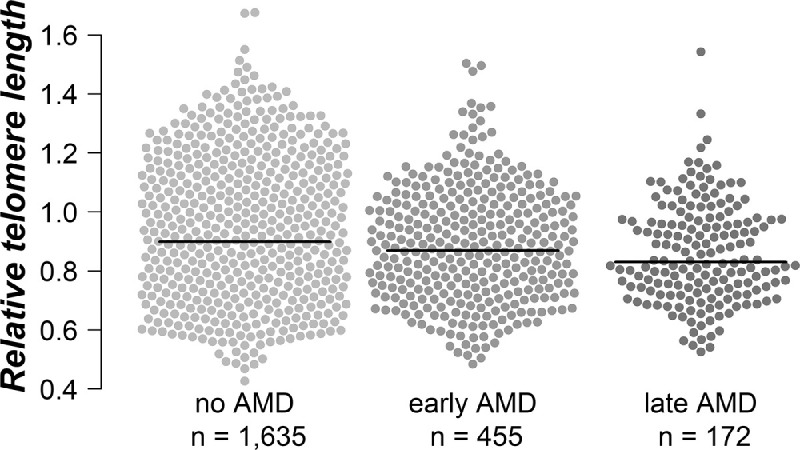
Comparison of RTL among individuals without AMD (*n* = 1635) and those with early (*n* = 455) or late AMD (*n* = 172). The *black lines* represent the non-adjusted median of the relative telomere length of each group.

At baseline, the AugUR study—with given AMD status and available DNA—includes 172 participants with late AMD (including 98 with neovascular AMD, 37 with geographic atrophy, and 37 with a combined subtype) and 455 participants with early AMD according to the Three Continent AMD Consortium Severity Scale.^9^ These 627 individuals with AMD and 1635 participants without AMD were included in the present analyses. The baseline characteristics of all participants are provided in Table 1. We observed significant differences between individuals with and without AMD in the following parameters: age, triglycerides, high-density lipoprotein (HDL) cholesterol, estimated glomerular filtration rate, diastolic blood pressure, hand grip strength, and RTL. Supplementary Table S1 provides additional baseline characteristics for participants without and those with early and late AMD. A highly significant difference (*P* < 0.001) for RTL was found among the three groups, with the longest RTL in the individuals without AMD (0.91 ± 0.18), the shortest RTL in persons with late AMD (0.86 ± 0.17), and RTL in between the two groups for those with early AMD (0.88 ± 0.17) (Fig. 1).

**Table 1.** Baseline Characteristics of Individuals Without AMD and Individuals With Any AMD (Early AMD According to the Three Continent AMD Consortium Severity Scale or Late AMD)

	No AMD (*n* = 1635)	Any AMD (*n* = 627)	*P*
Sex, *n* (% male)	796 (48.7)	281 (44.8)	0.10
Age (y), mean ± SD (25th; 50th; 75th percentile)	77.6 ± 4.7 (74.0; 76.8; 80.6)	79.9 ± 5.1 (75.6; 79.3; 83.6)	**<0.001**
Smoking status, *n* (%)			0.53
Current	90 (5.5)	36 (5.8)	
Former	644 (39.5)	230 (36.9)	
Never	897 (55.0)	357 (57.3)	
Body mass index (kg/m²), mean ± SD (25th; 50th; 75th percentile)	27.7 ± 4.5 (24.7; 27.2; 30.4)	27.7 ± 4.6 (24.8; 27.1; 30.0)	0.97
Diabetes mellitus, *n* (%)	334 (20.4)	126 (20.1)	0.86
Waist-to-hip ratio, mean ± SD (25th; 50th; 75th percentile)	0.95 ± 0.09 (0.89; 0.95; 1.01)	0.95 ± 0.09 (0.88; 0.94; 1.01)	0.83
Alcohol (g/d), mean ± SD	10.1 ± 12.4	9.3 ± 10.5	0.17
Extreme light exposure in the past, *n* (%)	122 (7.5)	39 (6.3)	0.32
Triglycerides (mg/dL)	160 ± 84 (102; 140; 195)	152 ± 87 (93; 134; 183)	**0.04**
Cholesterol (mg/dL), mean ± SD (25th; 50th; 75th percentile)			
LDL	142 ± 35 (116; 141; 164)	140 ± 34 (116; 139; 163)	0.20
HDL	60.7 ± 15.2 (49.6; 59.0,69.8)	62.4 ± 16.3 (50.9; 61.7; 71.3)	**0.02**
Total	218 ± 46 (185; 218; 248)	217 ± 46 (185; 216; 249)	0.70
C-reactive protein (mg/dL),^*^ mean ± SD (25th; 50th; 75th percentile)	0.43 ± 0.56 (0.21; 0.21; 0.42)	0.47 ± 0.79 (0.21; 0.21; 0.43)	0.19
HbA_1c_ (mmol/mol), mean ± SD (25th; 50th; 75th percentile)	39.9 ± 7.3 (35.5; 38.4; 42.1)	39.5 ± 7.6 (35.1; 37.7; 41.9)	0.34
eGFR (mL/min/1.73 m^2^), mean ± SD	68.7 ± 15.9	66.0 ± 17.4	**<0.001**
Blood pressure (mmHg), mean ± SD (25th; 50th; 75th percentile)			
Systolic	132 ± 18 (119; 131; 143)	131 ± 18 (119; 131; 142)	0.82
Diastolic	77 ± 11 (70; 77; 84)	75 ± 10 (69; 75; 82)	**0.004**
Hypertension,^†^ *n* (%)	1178 (72.1)	458 (73.2)	0.63
Cardiovascular disease,^‡^ *n* (%)	345 (21.3)	140 (22.5)	0.53
Medication intake, *n* (%)	1576 (96.6)	607 (97.4)	0.30
Hand grip strength (kg), mean ± SD (25th; 50th; 75th percentile)	30.7 ± 9.9 (23.0; 28.8; 38.3)	29.0 ± 9.5 (21.8; 27.0; 35.6)	**<0.001**
Relative telomere length, mean ± SD (25th; 50th; 75th percentile)	0.91 ± 0.18 (0.78; 0.90; 1.03)	0.88 ± 0.17 (0.76; 0.86; 0.98)	**<0.001**

Statistically significant p values are presented in bold.

LDL, low-density lipoprotein; CRP = C-reactive protein; eGFR = estimated glomerular filtration rate.

* CRP has a lower limit of detection (LOD) of <0.3, which was replaced by the value = LOD/sqrt(2).

† Hypertension was defined by systolic blood pressure ≥ 140 mmHg and/or diastolic blood pressure ≥ 90 mmHg and/or the use of antihypertensive drugs.

‡ Cardiovascular disease was defined as a history of myocardial infarction, stroke, and/or presence of coronary stents or bypass.

### Relative Telomere Length and AMD

**Figure 2.**
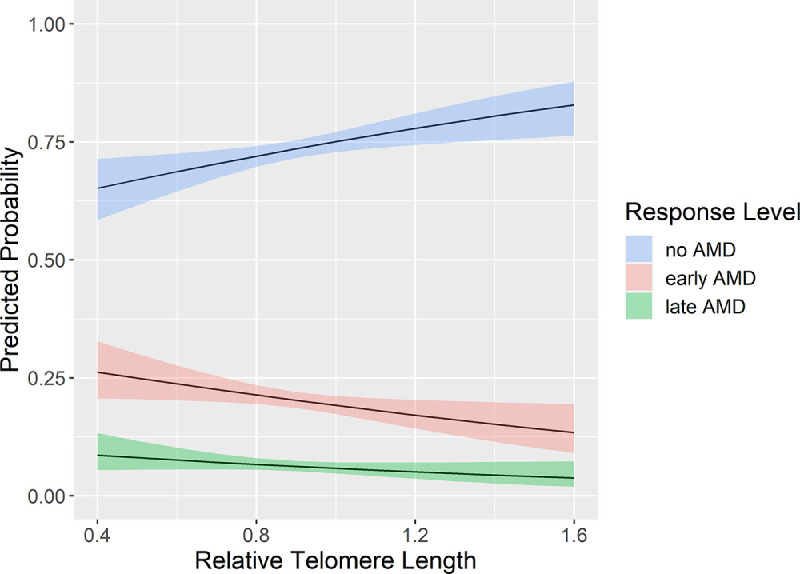
Effect plot depicting the predicted probability (with 95% CIs; *y*-axis) of having no AMD (*n* = 1635), early AMD (*n* = 455), or late AMD (*n* = 172), given a specific relative RTL (*x*-axis), adjusted for age and sex.

We started with age- and sex-adjusted multinomial regression analyses to investigate the association of RTL and AMD. Per an RTL decline of 0.1 unit, we observed an OR of 1.08 (95% CI, 1.01–1.15; *P* = 0.01) and OR = 1.09 (95% CI, 0.99–1.20; *P* = 0.07) for the risk for early and late AMD, respectively. Additionally, we calculated the predicted probabilities for a given RTL to have no, early, or late AMD based on multinomial regression models (adjusted for age and sex). Figure 2 shows that increasing RTL increased the probability to be free of AMD, whereas increased RTL decreased the probability for both early and late AMD in the same parallel manner. For example, the predicted probability of a person with RTL 0.4 was 26% to have early AMD, 9% to have late AMD, and 65% to be free of AMD (Supplementary Table S2). A person with a RTL of 1.6 has only half of the risk for both early (13%) and late AMD (4%). Altogether, with decreasing RTL, the probability to have AMD increases. The likelihood ratio test supported our hypothesis of an association of RTL with AMD and highlighted that the predictor variable RTL adds significant value to the model (*P* = 0.017, age- and sex-adjusted).

Based on these results, we combined early and late AMD into one group and used logistic regression models for “any AMD” as the outcome for all further evaluations for the entire sample, but we also stratified for women and men (Table 2). A decline by 0.1 RTL unit increased the probability for AMD by 8% in the age- and sex-adjusted model (OR = 1.08; 95% CI, 1.02–1.14; *P* = 0.005). This association remained stable when adding other risk factors (smoking, HDL cholesterol, diabetes, cardiovascular disease, and hypertension) for AMD, supporting an independent association between RTL and AMD (see fully adjusted model 5 in Table 2). The risk estimate per 0.1 RTL unit decrease on AMD is comparable to the estimate of a 1-year increase in age (OR = 1.09; 95% CI, 1.07–1.11; *P* < 0.001).

**Table 2.** Logistic Regression Analysis Investigating the Association of RTL (Decrease by 0.1) and AMD Using Various Adjustment Models^*^

	All (*N* = 2262)		Women^†^ (*n* = 1185)		Men^†^ (*n* = 1077)	
RTL	OR (95% CI)	*P*	OR (95% CI)	*P*	OR (95% CI)	*P*
Model 1 unadjusted	1.13 (1.07–1.19)	<0.001	1.19 (1.10–1.28)	<0.001	1.07 (0.99–1.16)	0.08
Model 2 adjusted for sex	1.13 (1.08–1.20)	<0.001	—		—	
Model 3 adjusted for age and sex	1.08 (1.02–1.14)	0.005	1.14 (1.06–1.23)	<0.001	1.01 (0.93–1.10)	0.76
Model 4 adjusted for age, sex, and current smoking	1.08 (1.02–1.14)	0.007	1.14 (1.05–1.22)	<0.001	1.01 (0.93–1.10)	0.76
Model 5 adjusted for age, sex, current smoking, HDL cholesterol, diabetes, cardiovascular disease, and hypertension	1.07 (1.01–1.14)	0.016	1.12 (1.04–1.22)	0.003	1.02 (0.93–1.11)	0.73

* *N* refers to the participants with available RTL measurements and AMD classification status. Of the 2262 participants, 1635 showed no signs of AMD, and 627 participants had either early or late AMD according to the Three Continent AMD Consortium Severity Scale.

† Sex-specific models are not adjusted for sex.

Interestingly, the effect of the RTL on AMD was only present in women (OR = 1.14; 95% CI, 1.06–1.23; *P* < 0.001) and not in men (OR = 1.01; 95% CI, 0.93–1.10; *P* = 0.76) in the age-adjusted logistic regression model. This sex-dependent effect was not influenced by different adjustment models. A formal test revealed a significant sex-by-RTL interaction on AMD (*P* = 0.043). The distribution of AMD status and most other relevant parameters was similar in both sexes (Supplementary Table S3). Supplementary Table S4 provides additional sensitivity analyses with further adjustments for several potential confounders and risk factors, including extreme light exposure and cardiovascular parameters, in the entire cohort and stratified for men and women, which did not change our main findings.

**Figure 3.**
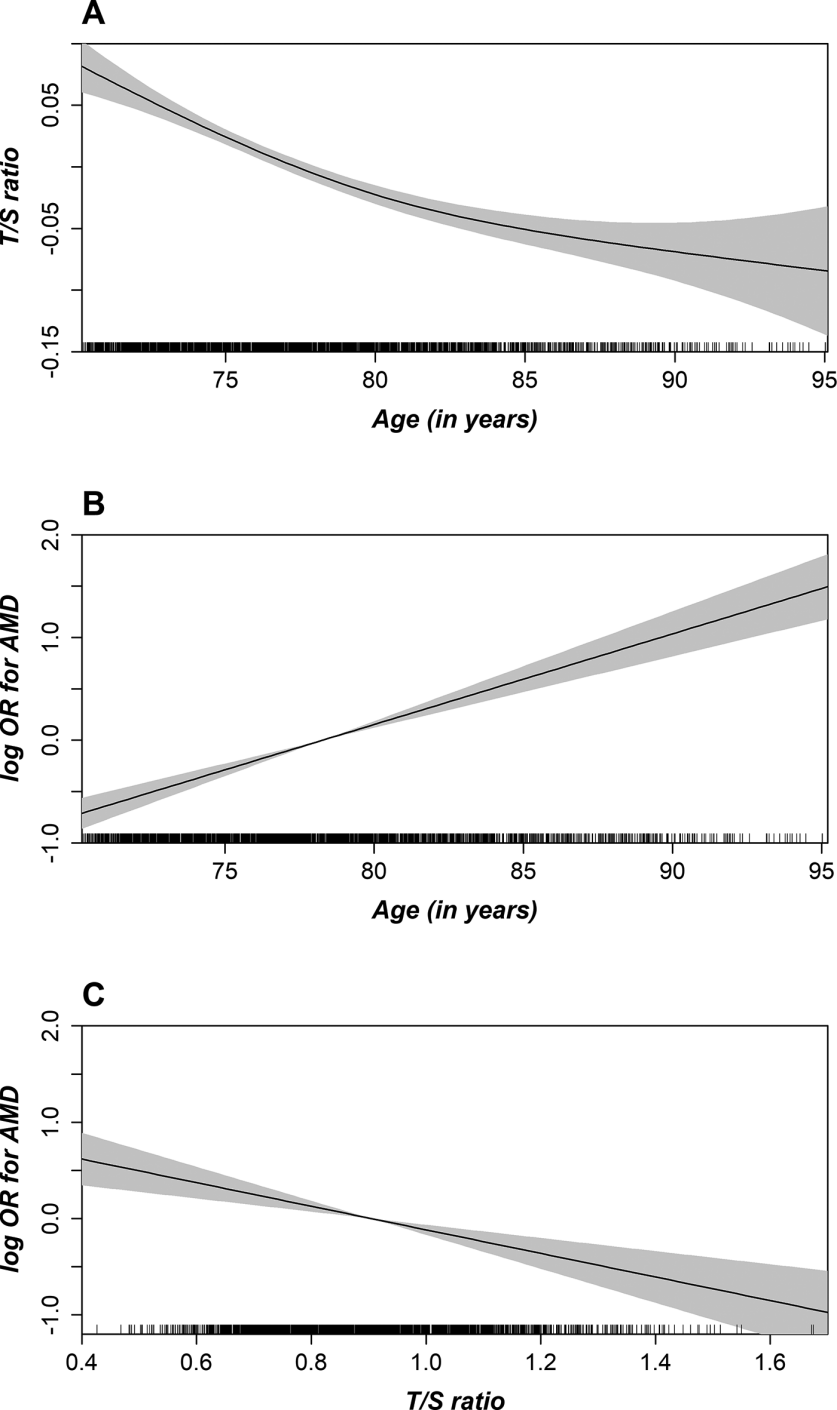
Unadjusted spline with 95% confidence bands demonstrating the linear regression between relative telomere length and age (**A**), the association between AMD and age (**B**), or the RTL (**C**). On the *y*-axis, the logarithmic (log) ORs for AMD are plotted against age in years (**B**) or the RTL as the relative telomere length (**C**) on the *x*-axis (*n* = 2262).

Moreover, splines for linear and logistic regression were created to investigate the association between age and RTL (Fig. 3A), age and AMD (Fig. 3B), or RTL and AMD (Fig. 3C). Figure 3A illustrates a decline of RTL with increasing age. Figure 3B demonstrates a linear increase of the AMD risk with age independent from adjustment variables and thereby justifies treating age as a linear variable in the initial regression models. Figure 3C shows the highest risk for AMD with the shortest RTL.

## Discussion

In the study at hand, we found RTL to be significantly lower in individuals with AMD compared to AMD-free participants. Each 0.1 unit shorter RTL increased the odds for AMD by 8% in the age- and sex-adjusted model, and the association remained stable and significant in all other models adjusting for further AMD risk factors. A sex-stratified analysis revealed that this association was solely present in women and not in men (OR = 1.14 vs. OR = 1.01, respectively).

### Relative Telomere Length and AMD

Up to now, only a few, mostly small, studies have investigated the association of RTL with AMD with conflicting results. A Han Chinese case-control study (197 patients with advanced AMD and 259 controls) measured RTL by qPCR and found shorter telomere length to be associated with an increased odds for AMD (OR = 2.24; 95% CI, 1.68–3.07; *P* < 0.001) in an age- and sex-adjusted model.^28^ This is directionally in line with our results, but with larger effect. The differences in age were very high between patients and controls (mean ≈ 75 vs. 68 years), and it is unclear whether a simple adjustment for age was able to control for this important confounder. Furthermore, patients and controls were neither characterized nor controlled for any other AMD risk factors and confounders.^28^ A most recent study from Lithuania has found opposite results in 56 cases with late AMD (geographic atrophy) and 73 AMD-free control individuals. They found a tremendously increased RTL in patients with AMD compared to controls (mean T/S ratio = 1.64 vs. 0.76; *P* < 0.001 in an age- and sex-adjusted model) without controlling for other confounders.^30^ The reason for this pronounced difference is elusive. In a third study from Finland, no association for RTL between 121 advanced AMD patients and 77 AMD-free controls was observed, but also no association for typical AMD risk factors with AMD, which could be attributed to insufficient power.^29^

An entirely different approach has been applied using 1955 UK Biobank individuals who self-reported AMD and 84,778 controls. The authors did not directly measure RTL but used nine single nucleotide polymorphisms as instrumental variables that genetically predicted telomere length.^36^ They found no association between genetically increased telomere length and AMD (OR = 1.02; 95% CI, 0.68–1.53), which might be explained by several reasons: First, the instrumental variable for telomere length explained only a minor fraction (2%–3%) of telomere length variability; second, participants in the UK Biobank are 40 to 69 years of age, an age group in which AMD rarely occurs; and, third, AMD was assessed only by a questionnaire instead of imaging and AMD grading by an experienced ophthalmologist, as was done in our study.^36^

### Biological Hypothesis

Age-related macular degeneration is clearly associated with inflammation.^1^^,^^10^^–^^12^ Components and genes of the complement cascade,^37^ the extracellular matrix, and the lipid metabolism^38^^,^^39^ are well-investigated genetic risk factors of this disease. In this context, oxidative stress appears to be particularly relevant, as telomeres contain a high amount of the nucleotide guanine, which is more likely to be oxidized than other DNA bases. Several AMD risk genes are known to encode for proteins associated with oxidative stress.^17^^,^^40^^,^^41^

It is not fully clarified to what extent oxidative stress influences the development of AMD. Premature telomere shortening and induction of senescence or cell death can be triggered by oxidative stress via the activation of p53. Consequently, ROS levels increase, and further DNA damage of both mitochondrial and nuclear DNA appears.^17^^,^^20^^,^^40^^,^^41^ The p53 activation induced by telomere shortening represses the peroxisome proliferator-activated receptor gamma coactivator (PGC), a major regulator of mitochondria.^42^ Generally, PGC-1α reduces oxidative stress and senescence and in contrast supports proteostasis and telomerase (reviewed in Reference 43). Telomerase is known to compensate the telomere shortening, and, in mitochondria, it has a positive effect on mitochondrial DNA repair and the reduction of excessive ROS.^44^^,^^45^ This supports the hypothesis that altered telomere length and consequently altered mitochondrial (dys)function are co-dependent and relevant for AMD development.

However, the influence of oxidative stress on telomere shortening in vivo might be more complex. It is hypothesized that telomere attrition is induced when the energy household of a cell is unbalanced and the anabolic metabolism has to be reduced via the target of rapamycin.^46^^–^^48^ Interestingly, there is evidence of alterations in the photoreceptor metabolism in AMD.^49^ Additionally, the target of rapamycin kinase is essential in aging and, moreover, a potential therapeutic target for AMD.^50^

### Interaction with Sex

We observed a strong sex-dependent association of RTL with AMD, as the association was solely present in women. Telomere dynamics is associated with biological sex, and, in general, women have longer telomeres compared to men,^51^^–^^53^ which was also the case in our study (0.915 in women vs. 0.892 in men; *P* = 0.003). It has been shown that oxidative stress influences the rate of telomere shortening.^20^ Due to their higher estrogen levels, women are reported to have lower levels of ROS and higher telomerase activity.^53^^,^^54^ In the AugUR study, most participant characteristics were similarly distributed between men and women with some exceptions. However, the adjustment for all of these factors had no effect on the sex-dependent association of RTL with AMD in our study. This is true even for parameters with strong differences in the descriptive data, such as cardiovascular disease, smoking status, or extreme light exposure in the past. Although some of these factors are associated with RTL,^34^^,^^35^^,^^55^ none of them causes the sex-specific differences in the effect of RTL on AMD we have reported here.

Even though AMD is driven mainly genetically (∼47%),^39^ a recent genome-wide association study revealed no novel genome-wide significant loci with sex differences, and none of the known AMD loci showed significant sex differences.^56^ The role biological sex has for AMD is highly debated, and, although some studies have reported an association, most studies could not confirm these findings.^5^^,^^55^ Our results suggest a sex-differential pathway potentially via sex-specific ROS and telomerase activity on oxidative stress–related causes of AMD. More studies including parameters such as hormonal differences are needed to further understand the association of RTL in AMD in the context of sex.

### Biological and Chronological Age

One of the most critical points in the context of etiological factors for AMD is the disentangling of the relationship between age and RTL with regard to their effect on AMD. There might be an interaction, and they could be predictors, confounders, or proxy variables for each other. Relative telomere length might reflect the biological age, which is not necessarily identical with the chronological age of an individual. However, on a genetic basis, there is no overlap between the risk genes of AMD (e.g., *CFH*, *C2*, *C3*, *CETP*, *VEGFA*, *APOE*)^37^^,^^57^ and the genes that have an influence on advanced shortening of the telomeres (e.g., *TERT*, *TERC*, *TRF1*, NAF1, RTEL).^30^^,^^58^^,^^59^ We employed different adjustments for age to eliminate age as confounder (see Supplementary Materials). Our results suggest an age-independent association between RTL and AMD. In our analyses, the association of RTL on AMD is stronger when not adjusted for chronological age (Table 2, model 1). Therefore, the importance of considering chronological age versus biological age separately in medical research could be discussed. Notably, RTL in general is associated with several (age-related) diseases and is not eligible as a marker specific to AMD.

In addition, one could speculate whether this elderly study sample represents a cohort more resilient to telomere shortening than those generally older in age, considering that only the mobile (physically and mentally fit) elderly participated in the study. Therefore, these participants could be the ones more resistant to telomere attrition compared to those not participating due to frailty, severe disease, or earlier succumbing to a disease.

### Strengths and Limitations of the Study

It is a strength of the study that all participants were recruited and examined via the same standardized procedures, and AMD grading was performed by an experienced ophthalmologist. All participants were above 70 years old and from the same geographical area. To avoid batch effects, all DNAs were prepared and sent to our laboratory at the same time. All RTL measurements were performed under the exact same conditions, with identical reagents and within a short period of time. Most importantly, the laboratory personnel were blinded with regard to the AMD status, and samples were randomly distributed within each assay run. Finally, to our knowledge, this is the largest investigation of RTL measurements and AMD.

AMD grading based on color fundus photography is a well-established approach widely used in epidemiological studies. Optical coherence tomography might be more sensitive and has only recently been implemented in epidemiological studies. We acknowledge the potential limitation of color fundus photography (e.g., false-negative results), but our assessment of intergrader variability documented a high reproducibility. As this study includes only elderly individuals, almost all participants (>96%) reported medication intake and most of them had comorbidities, which makes the comparison and the identification of predictors of the disease of interest more difficult. Because the study is of a cross-sectional nature, we cannot disentangle whether shorter telomeres increase the risk of developing AMD or whether they are purely a marker of biological aging beyond chronological age. It might even be possible that shorter telomeres are a consequence of AMD or AMD-related processes. Longitudinal studies with serial measurements of RTL and AMD are required to further evaluate this association. The further investigation of biological versus chronological age and further insights of the sex-differential RLT-linked pathway between oxidative stress and AMD may help us understand the underlying processes behind this association.

## Conclusions

In this study, we found an association between shorter RTL and AMD in an elderly population independent from potential risk factors. This association was present only in women and not in men, driven by biological age and to some extent independent from chronological age. Our results are in line with a sex-differential pathway from oxidative stress to AMD. Further studies on the progression of the disease including follow-up data are required for a better understanding of the pathomechanisms of AMD.

## Supplementary Material

Supplement 1
