# Supplementary material for: Relative Telomere Length Is Associated With Age-Related Macular Degeneration in Women
Source: Invest Ophthalmol Vis Sci. 2022 May 25;63(5):30. doi: 10.1167/iovs.63.5.30 (PMC9150829; doi:10.1167/iovs.63.5.30)
Supplement: Supplement 1 [file iovs-63-5-30_s001.pdf]

Supplemental Material for

Relative telomere length is associated with age-related macular degeneration in women

Adriana Koller [1], Caroline Brandl [2,3], Claudia Lamina [1], Martina E. Zimmermann [2], Monika Summerer [1], Klaus J. Stark [2], Reinhard Würzner [4], Iris M. Heid [2], Florian Kronenberg [1]

[1] Institute of Genetic Epidemiology, Medical University of Innsbruck, Innsbruck, Austria

[2] Department of Genetic Epidemiology, University of Regensburg, Regensburg, Germany

[3] Department of Ophthalmology, University Hospital Regensburg, Regensburg, Germany

[4] Institute of Hygiene and Medical Microbiology, Medical University of Innsbruck, Innsbruck, Austria

SUPPLEMENTARY METHODS

Study Population

The ongoing AugUR study includes over 2,400 probands from the mobile elderly population of Regensburg with at least 70 years of age. For the baseline investigation of the AugUR study, a random sample from the local population registries of residence was invited via mail. Of the 13,522 individuals who received a study invitation via mail, 5,351 responded and of those, 2,449 participated in the AugUR study (response rate=39.6%, participation rate=18.1%). Reasons for non-participation were being too sick, lack of time, no interest in participating or others (Supplementary Figure S1). No a priori exclusion criteria were defined. Those interested in participating in the study were contacted and an appointment was made. All participants came to the study center and were mentally and physically mobile to conduct the protocol (face-to-face interview, blood draw and further medical and ophthalmological investigations). The recruitment of participants has taken place between 2013 and 2019. Relative telomere length measurements were performed in May 2021 in DNA collected at the time of the baseline and ophthalmological investigation.

During the first medical interview, information on sociodemographic data, lifestyle factors, metabolic parameters, medication use, general comorbidities and ocular comorbidities, e.g., cataract, glaucoma and diabetic retinopathy was collected. In addition to the detailed ophthalmological investigations and medical interviews, both, blood and urine samples were collected.[1,2]

Variable ascertainment and definitions

Smoking status was obtained as current smoker (≥1 cigarette per month), ex-smokers (quitted smoking at least a month ago) and never smokers (smokes <100 cigarettes in their lifetime). All lipid blood parameters were assessed under non-fasting conditions. Hypertension was defined by systolic blood pressure ≥140 mmHg and/or diastolic blood pressure ≥90 mmHg and/or the use of antihypertensive drugs. Diabetes status was based on self-reported diabetes and additional diabetes medication. Cardiovascular disease was defined as a history of myocardial infarctions, stroke and/or presence of coronary stents or bypass. The estimated glomerular filtration rate was calculated based on the 2009 CKD-EPI equation.[3] Information on extreme light exposure was gathered in a questionnaire asking whether the participants were working predominantly outdoors or exposed to extreme light at age 30 to 50 years. Finally, general fitness was assessed using hand grip strength (Jamar Plus+ Digital Hand Dynamometer; Patterson Medical, Warrenville, IL, USA). Each hand was tested three times and the maximum value of the six measurements (in kg) was analyzed.

DNA extraction

It has been described previously that the method of DNA extraction greatly influences the results of relative telomere length (RTL) measurements [4,5] The DNA extraction of the first 959 samples was done using the protocol and reagents from Puregene (Qiagen, Hilden, Germany). However, due to high fluctuations in DNA concentrations and narrow yield in this elderly cohort, the procedure for the remaining 1,442 samples changed to another, however, very similar salting out method. Here, all buffers were manually prepared. DNA concentration and purity were checked using a Tecan Infinite M200pro (Tecan Group Ltd., Männedorf, Switzerland) and diluted with water to a final concentration of 20 ng/µl. To investigate whether the slightly different DNA extraction methods influence the measurements of the RTL, DNAs from 20 whole blood samples were extracted with both methods. However, these results correlated perfectly (correlation coefficient=0.96, $p<0.001$) and therefore, both subsets in the AugUR study were analyzed as one cohort.

Relative telomere length measurements

In contrast to the previously used singleplex method, using a multiplex approach allows the amplification and detection of both products, the telomere product and the single-copy gene product (albumin), within one run. Therefore, this approach reduces the error-proneness, is cheaper and less time-consuming.[6,7] Primers were designed by Cawthon *et al*.[6] with an update received by personal communication with the author and synthesized by Microsynth AG (Balgach, Switzerland). Exact primer sequences starting with 5´ were

- telg (ACACTAAGGTTTGGGTTTGGGTTTGGGTTTGGGTTAGTGT),
- telc (TGTTAGGTATCCCTATCCCTATCCCTATCCCTATCCCTAACA),
- albugcr1 (CGGCGGCGGGCGGCGCGGGCTGGGCGGAAACGCTGCGCAGAATCCTTG) and
- albdgcr1 (GCCCGGCCCGCCGCGCCCGTCCCGCCGCTGAAAAGTACGGTCGCCTG).

The cycling profile was optimized to improve efficiencies: 1x stage one (95°C, 15 min), 2x stage two (94°C for 15 sec, 49°C for 15 sec), 40x stage three (94°C for 15 sec, 68°C for 10 sec, 74°C for 15 sec with signal acquisition, 82°C for 10 sec, 88°C for 15 sec with signal acquisition). A melting curve (59-95 °C with 5 seconds per step, 0.5 °C each) was included after each run.

To investigate the efficiency of the assay, a three-fold serial dilution with five different concentrations was prepared starting with a concentration of 60 ng. For the standard curve, the same conditions as in the final assay were chosen, however, reactions were performed in quadruplicates instead of triplicates. Efficiencies above 90 % for both targets were detected. Each 10 µl reaction contained 1 µl DNA (20 ng), 5 µl PowerUp™ SYBR™ Green Master Mix (Thermo Fisher Scientific, Waltham, MA, USA),

700 nM telomere primers each as well as 400 nM albumin primers each. All qPCR reactions were run on a Quantstudio 6 flex system (Software 1.7.1, Thermo Fisher Scientific, Waltham, MA, USA).

Before calculation, the efficiency correction method[8] was applied on the raw data for each well to calculate an individual PCR efficiency using the software LinRegPCR [9] for both targets individually. Using the three efficiencies and Ct values per sample and per product (telomere or albumin), a coefficient of variation below 5% was set as a requirement to keep the sample. Next, the mean of the triplicates for each sample was calculated. To determine the relative T/S ratio that reflects the difference between the samples normalized to the standard, the following formula was chosen to calculate the fraction efficiency (eff) of the samples to the Ct value of the samples over the fraction of the efficiency of the calibrator to the Ct value of the calibrator. With this method, the relative telomere length is determined via the ratio between the telomere content and the nuclear reference gene content.

$$Relative\,T/S\,Ratio = \frac{eff(tel, sample)^{Ct(tel,sample)}}{eff(alb, sample)^{Ct(alb,sample)}} \Big/ \frac{eff(tel, calibrator)^{Ct(tel,calibrator)}}{eff(alb, calibrator)^{Ct(alb,calibrator)}}$$

For calculation of the T/S ratio, an intern control was chosen as a calibrator, defined as T/S ratio=1. All other T/S ratios were calculated based on the calibrator and therefore, for each sample a ratio larger (>1) or smaller (<1) than the one of the calibrator was determined. Four different positive controls were included as additional references: a manufactured DNA (commercially available DNA; Human Genomic DNA, Roche, Merck KGaA, Darmstadt, Germany) and three control samples used for comparison of the inter-assay variability. These four positive controls were included on all plates and their T/S ratios were compared for the entire study after finishing all measurements. To avoid bias of the calibrator, a plate correction factor was used in order to correct the T/S ratios for variability in the calibrator in plates where all four positive controls show a variability over 10%. After completing all runs in the study, the T/S ratios of all positive controls were compared and finally the plate correction factor was used in three of the twenty-six independent experiments. An inter-assay variability below 5% was determined for each of the four positive controls over the entire study.

Power calculation

A priori power calculations for a logistic regression model were performed based on the sample size available in the AugUR study (Supplementary Figure S2). The minimally detectable odds ratios are given per standard deviation difference of a normal distributed standardized variable. Since RTL is correlated with age, the power calculations are given once for "no adjustment for age" and once for "with adjustment for age" assuming a correlation with age of $r^2 = 0.60$ in the worst case.

Sensitivity analyses

Sensitivity analyses for the entire cohort but also stratified for men and women are provided in Supplementary Table S4. Since the age distribution was different for individuals with and without AMD, we performed a further sensitivity analysis by additionally adjusting not only for age but also for (age-median age)^2 and (age-median age)^3 in each model. No changes in the ß-estimates or the significance were detected.

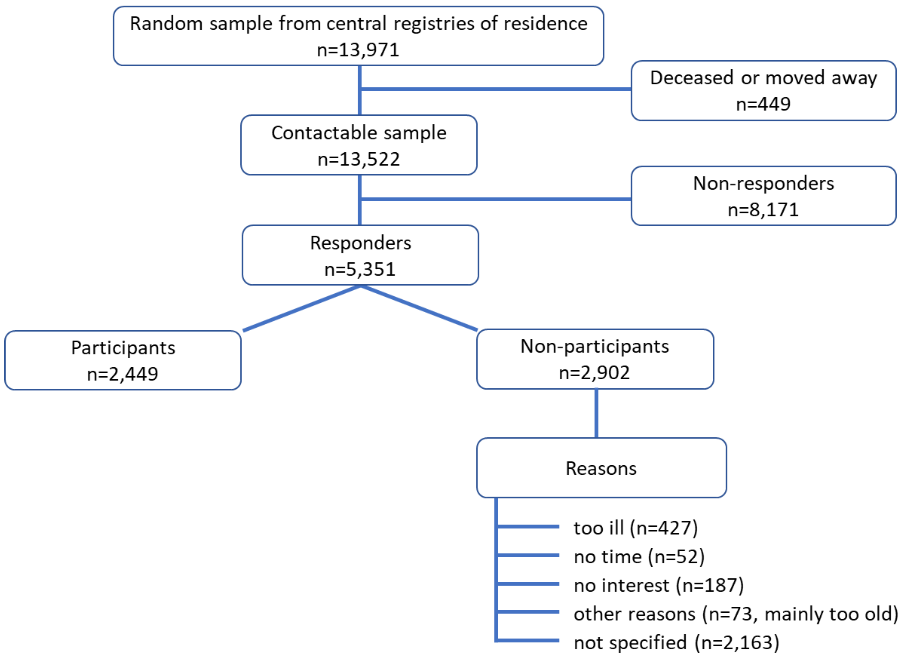

Supplementary Figure S1: Schematic overview of AugUR study recruitment phase from first contact to final participation at the baseline.

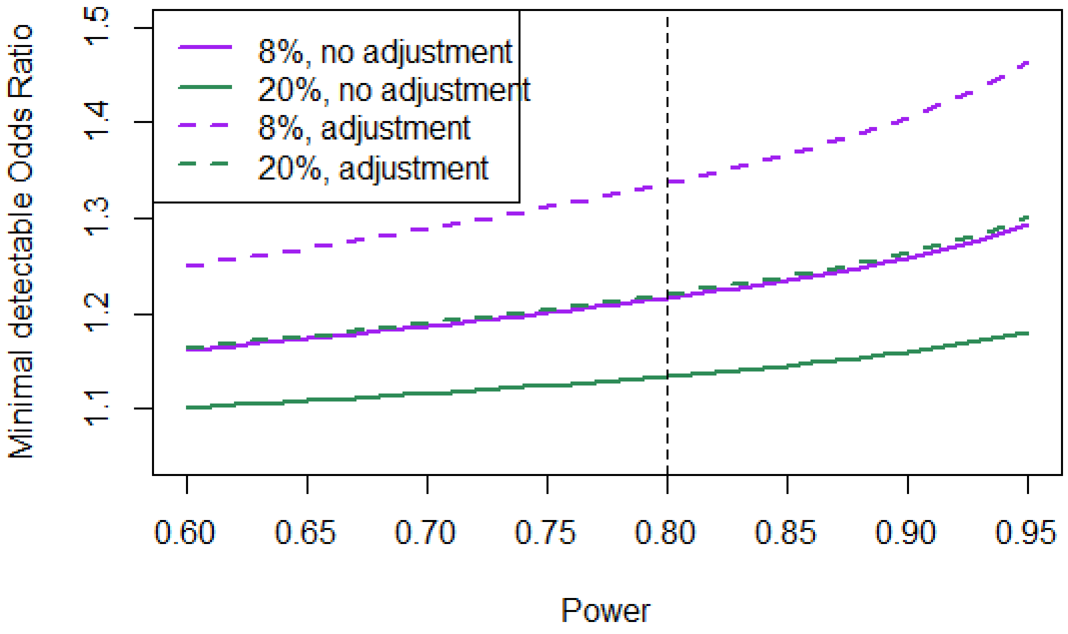

Supplementary Figure S2: *A priori* power calculation based on the observed prevalence of 8% for late AMD and 20% for early AMD according to the Three-Continent classification. The minimally detectable odds ratios are given per standard deviation difference of a normal distributed standardized variable and shown with (dashed lines) and without adjustment (solid line).

Supplementary Table S1: Baseline characteristics of individuals without AMD, with early AMD (according to Three Continent AMD Consortium Severity Scale) and participants with late AMD (n=2,262).

	**No AMD (n=1,635)**	**Early AMD (n=455)**	**Late AMD (n=172)**	***P*-value**
Sex, n (% male)	796 (48.7%)	200 (44.0%)	81 (47.1%)	0.20
Age (years)	77.6±4.7 [74.0; 76.8; 80.6]	79.2±5.1 [75.2; 78.6; 82.6]	81.9±5.7 [77.7; 81.6; 86.4]	**<0.001**
Smoking n (%)				0.11
Current	90 (5.5%)	20 (4.4%)	16 (9.4%)	
Former	644 (39.5%)	173 (38.2%)	57 (33.5%)	
Never	897 (55.0%)	260 (57.4%)	97 (57.1%)	
Body Mass Index (kg/m^2)	27.7±4.5 [24.7; 27.2; 30.4]	27.6±4.7 [24.8; 27.0; 29.8]	28.1±4.4 [25.4; 27.7; 30.3]	0.57
Diabetes Mellitus, n (%)	334 (20.4%)	83 (18.2%)	43 (25.0%)	0.17
Waist-hip ratio	0.95±0.09 [0.89; 0.95; 1.01]	0.94±0.09 [0.88; 0.94; 1.00]	0.96±0.09 [0.90; 0.96; 1.02]	0.06
Alcohol (g per day)	10.1 ±12.4	9.8±10.6	8.1±10.0	0.12
Extreme light exposure in the past, n (%)	122 (7.5%)	27 (6.0%)	12 (7.1%)	0.54
Triglycerides (mg/dl)	160±84 [102;140;195]	148±84 [92;129;183]	160±92 [97;142;187]	**0.04**
LDL cholesterol (mg/dl)	142±35 [116; 141; 164]	140±34 [116; 139; 162]	139±35 [115; 140; 163]	0.43
HDL cholesterol (mg/dl)	60.7±15.2 [49.6; 59.0,69.8]	63.6±16.6 [52.1; 61.7,72.7]	59.5±15.1 [47.7; 58.7;68.9]	**<0.001**
Total cholesterol (mg/dl)	218±46 [185; 218; 248]	218±48 [186; 216; 249]	218±45 [184; 216; 249]	0.75
C-reactive protein (mg/dl)*	0.43±0.56 [0.21;0.21; 0.42]	0.43±0.71 [0.21;0.21; 0.41]	0.57±0.98 [0.21;0.21; 0.59]	**0.008**
HbA_{1c} (mmol/mol)	39.9±7.3 [35.5; 38.4; 42.1]	38.9±6.8 [34.5; 37.7; 41.0]	41.2±9.4 [35.5; 38.8; 45.4]	**0.003**
eGFR (mL/min/1.73m^2)	68.7±15.9	67.2±16.0	63.1±20.4	**<0.001**
Systolic blood pressure (mmHg)	132±18 [119; 131; 143]	131±18 [119; 130; 141]	133±18 [121; 132; 144]	0.46
Diastolic blood pressure (mmHg)	77±11 [70; 77; 84]	75±10 [68; 75; 82]	76±10 [69; 76; 82]	**0.016**
Hypertension, n (%) †	1178 (72.1%)	326 (71.8%)	132 (76.7%)	0.41
Cardiovascular disease, n (%) ‡	345 (21.3%)	87 (19.3%)	53 (31.0%)	**0.006**
Medication intake, n (%)	1576 (96.6%)	439 (97.1%)	168 (98.2%)	0.45
Hand grip strength (in kg)	30.7±9.9 [23.0; 28.8; 38.3]	29.5±9.8 [22.1; 27.1; 36.7]	27.7±8.6 [21.3; 24.9; 33.7]	<0.001
Relative telomere length	0.91±0.18 [0.78/0.90/1.03]	0.88±0.17 [0.76/0.87;0.99]	0.86±0.17 [0.74/0.83/0.97]	**<0.001**

Abbreviations: AMD = age-related macular degeneration, LDL = low-density lipoprotein, HDL = high-density lipoprotein, CRP = C-reactive protein, eGFR = estimated glomerular filtration rate

Mean ± standard deviation [25th, 50th and 75th percentile] or number (%).

* CRP has a lower detection limit (LOD) of <0.3, which was replaced by the value = LOD/sqrt(2)

† Hypertension was defined by systolic blood pressure ≥140 mmHg and/or diastolic blood pressure ≥90 mmHg and/or the use of antihypertensive drugs.

‡ Cardiovascular disease was defined as a history of myocardial infarctions, stroke and/or presence of coronary stents or bypass.

Supplementary Table S2: Predicted probabilities with 95% confident intervals (CI) of no age-related macular degeneration (AMD) (n=1,635), early AMD (n=455) or late AMD (n=172), given a specific relative telomere length (adjusted for age and sex).

	No AMD		Early AMD		Late AMD	
**RTL**	**Predicted Probability**	**95% CI**	**Predicted Probability**	**95% CI**	**Predicted Probability**	**95% CI**
**0.40**	0.65	0.58-0.71	0.26	0.21-0.33	0.09	0.05-0.13
**0.80**	0.72	0.70-0.74	0.21	0.19-0.23	0.07	0.06-0.08
**1.20**	0.78	0.74-0.81	0.17	0.14-0.20	0.05	0.04-0.07
**1.60**	0.83	0.76-0.88	0.13	0.09-0.19	0.04	0.02-0.07

Supplementary Table S3: Comparison of baseline characteristics between men and women.

	**women (n = 1,185)**	**men (n = 1,077)**	***P*-value**
**AMD status, n (%)**			0.20
**No AMD**	839 (70.8%)	796 (73.9%)	
**Early AMD**	255 (21.5%)	200 (18.6%)	
**Late AMD**	91 (7.7%)	81 (7.5%)	
**Age (years)**	78.4±5.0	78.2±5.1	0.39
	[74.6; 77.7; 81.6]	[74.3; 77.2; 81.2]	
**Smoking status, n (%)**			<0.001
**Current**	55 (4.7%)	71 (6.6%)	
**Former**	285 (24.1)	589 (54.9)	
**Never**	841(71.2%)	413 (38.5%)	
**Body Mass Index (kg/m^2)**	27.6±5.0	27.8±3.	0.30
	[24.1; 27.0; 30.4]	[25.2; 27.4; 30.1]	
**Alcohol (g per day)**	6.2±7.8	13.9 ±14.1	<0.001
**Extreme light exposure in the past, n (%)**	8 (0.7%)	153 (16.6%)	<0.001
**Triglycerides (mg/dl)**	153±79	163±91	0.01
	[98;137;183]	[100;140;199]	
**LDL cholesterol (mg/dl)**	148.1±34.8	133.9±33.6	<0.001
	[123; 147; 169]	[109; 133; 154]	
**HDL cholesterol (mg/dl)**	66.4±15.1	55.4±13.7	<0.001
	[55.8; 65.3,75.7]	[45.8; 54.1,62.6]	
**Total cholesterol (mg/dl)**	230.8±45.0	203.7±42.4	<0.001
	[199; 231; 258]	[172; 202; 231]	
**HbA_{1c} (mmol/mol)**	39.8±7.4	39.8±7.4	0.83
	[35.5; 38.5; 41.8]	[35.5; 37.7; 42.1]	
**eGFR (mL/min/1.73m^2)**	68.0±16.5	67.8±16.2	0.83
**Systolic blood pressure (mmHg)**	130±18	133±17	<0.001
**Diastolic blood pressure (mmHg)**	76±11	76±11	0.68
**Hypertension, n (%)**	850 (71.9%)	786 (73.0%)	0.53
**Diabetes Mellitus, n (%)**	225 (19.0%)	235 (21.8%)	0.10
**Cardiovascular disease, n (%)**	154 (13.1%)	331 (31.1%)	<0.001
**Hand grip strength (in kg)**	23.1±5.0	38.1±7.5	<0.001
	[20.1; 23.0; 26.1]	[33.3; 38.1; 43.2]	
**Relative telomere length**	0.91±0.19	0.89±0.17	0.003
	[0.78/0.90;1.02]	[0.76/0.88/1.01]	

Abbrevations: see Table 1 in the main manuscript.

Supplementary Table S4: Additional sensitivity analyses for the logistic regression analysis investigating the association of RTL (decrease by 0.1 units) and AMD for entire study sample (n=2,262), women (n=1,185) and men (n=1,077) separately using different adjustment variables.

**Relative telomere length**	**All (n=2,262)**		**Women (n=1,185)**		**Men (n=1,077)**	
	**OR (95% CI)**	**p-value**	**OR (95% CI)**	**p-value**	**OR (95% CI)**	**p-value**
**Model 4*** (adjusted for age, sex, current smoking)	**1.08 (1.02-1.14)**	**0.007**	**1.14 (1.05-1.22)**	**<0.001**	**1.01 (0.93-1.10)**	**0.76**
as model 4 plus alcohol intake	1.08 (1.02-1.14)	0.009	1.13 (1.05-1.22)	<0.001	1.01 (0.93-1.10)	0.78
as model 4 plus extreme light exposure	1.08 (1.02-1.14)	0.008	1.13 (1.05-1.22)	<0.001	1.01 (0.93-1.10)	0.80
as model 4 plus total cholesterol	1.07 (1.02-1.14)	0.013	1.13 (1.05-1.23)	0.001	1.00 (0.92-1.09)	0.96
as model 4 plus triglycerides	1.07 (1.01-1.13)	0.019	1.13 (1.04-1.22)	0.003	1.01 (0.93-1.10)	0.86
as model 4 plus HDL-cholesterol	1.07 (1.01-1.13)	0.018	1.13 (1.05-1.22)	0.002	1.00 (0.92-1.09)	0.92
as model 4 plus LDL-cholesterol	1.07 (1.01-1.14)	0.016	1.13 (1.05-1.23)	0.001	1.00 (0.91-1.08)	0.91
as model 4 plus eGFR	1.08 (1.02-1.14)	0.008	1.13 (1.05-1.22)	0.001	1.01 (0.93-1.10)	0.75
as model 4 plus leukocyte count	1.08 (1.01-1.14)	0.011	1.13 (1.05-1.22)	0.002	1.01 (0.90-1.07)	0.74
as model 4 plus systolic blood pressure	1.08 (1.02-1.14)	0.007	1.14 (1.06-1.23)	<0.001	1.01 (0.93-1.10)	0.85
as model 4 plus diastolic blood pressure	1.07 (1.01-1.13)	0.007	1.14 (1.06-1.23)	<0.001	1.00 (0.92-1.09)	0.93
as model 4 plus HbA_{1c}	1.08 (1.01-1.13)	0.016	1.13 (1.04-1.22)	0.002	1.01 (0.93-1.10)	0.87
as model 4 plus cardiovascular disease	1.08 (1.01-1.13)	0.006	1.13 (1.05-1.22)	0.001	1.02 (0.94-1.11)	0.62
as model 4 plus hand grip strength	1.08 (1.03-1.15)	0.004	1.14 (1.06-1.23)	<0.001	1.02 (0.94-1.10)	0.71

*Model 4 is based on the logistic regression analyses presented in Table 2 of the main manuscript.

References

1. Stark K, Olden M, Brandl C, et al. The German AugUR study: study protocol of a prospective study to investigate chronic diseases in the elderly. *BMC Geriatr* 2015;15:130.
2. Brandl C, Zimmermann ME, Günther F, et al. On the impact of different approaches to classify age-related macular degeneration: Results from the German AugUR study. *Sci Rep* 2018;8(1):8675.
3. Levey AS, Stevens LA, Schmid CH, et al. A new equation to estimate glomerular filtration rate. *Ann Intern Med* 2009;150(9):604-12.
4. Cunningham JM, Johnson RA, Litzelman K, et al. Telomere length varies by DNA extraction method: implications for epidemiologic research. *Cancer Epidemiol Biomarkers Prev* 2013;22(11):2047-54.
5. Raschenberger J, Lamina C, Haun M, et al. Influence of DNA extraction methods on relative telomere length measurements and its impact on epidemiological studies. *Sci Rep* 2016;6:25398.
6. Cawthon RM. Telomere length measurement by a novel monochrome multiplex quantitative PCR method. *Nucleic Acids Res* 2009;37(3):e21.
7. Cawthon RM. Telomere measurement by quantitative PCR. *Nucleic Acids Res* 2002;30(10):e47.
8. Pfaffl MW. A new mathematical model for relative quantification in real-time RT-PCR. *Nucleic Acids Res* 2001;29(9):e45.
9. Ruijter JM, Ramakers C, Hoogaars WM, et al. Amplification efficiency: linking baseline and bias in the analysis of quantitative PCR data. *Nucleic Acids Res* 2009;37(6):e45.
